# A183 SECRETIONS OF LACTIC ACID BACTERIA AS ANTI-*HELICOBACTER PYLORI* AGENTS

**DOI:** 10.1093/jcag/gwad061.183

**Published:** 2024-02-14

**Authors:** C Creuzenet, D Carroll, J M Takougoum, m mohiuddin, J Auger, J McAlister, J Geddes-McAlister, S Bronner, S Binda

**Affiliations:** Microbiology and Immunology, Western University Schulich School of Medicine & Dentistry, London, ON, Canada; Microbiology and Immunology, Western University Schulich School of Medicine & Dentistry, London, ON, Canada; Microbiology and Immunology, Western University Schulich School of Medicine & Dentistry, London, ON, Canada; Microbiology and Immunology, Western University Schulich School of Medicine & Dentistry, London, ON, Canada; Rosell Institute for Microbiome and Probiotics, Lallemand Health Solutions, Montreal, QC, Canada; University of Guelph, Guelph, ON, Canada; University of Guelph, Guelph, ON, Canada; Rosell Institute for Microbiome and Probiotics, Lallemand Health Solutions, Montreal, QC, Canada; Rosell Institute for Microbiome and Probiotics, Lallemand Health Solutions, Montreal, QC, Canada

## Abstract

**Background:**

*Helicobacter pylori* (HP) colonizes chronically 50% of the world population, leading to gastric ulcers or cancers in 2-10 % of infections. Due to rising antibiotic resistance, curtailing HP-induced diseases requires the identification of new anti-HP molecules that can reduce HP viability or virulence. Several studies suggest that oral lactic acid bacteria (LAB) supplementation can enhance HP eradication treatment efficacy, suggesting that some LAB produce bioactive molecules with anti-HP properties. We previously found that 18 LAB secrete molecules with activity against two HP strains. The LAB secretions inhibited HP growth and reduced its urease activity, motility and ability to induce the pro-inflammatory cytokine IL-8 from gastric cells. This is important since gastric inflammation is the hallmark of HP infection.

**Aims:**

We aim to elucidate the mechanism of action involved using 5 LAB strains commercialized by Lallemand Health Solutions, including 2 from their Lacidofil^®^ probiotic formulation. Our goal is to harness the potential of the LAB-secreted molecules as novel alternatives to antibiotics or anti-inflammatory agents to curtail HP-induced gastric disease and concentrated cocktails of LAB secretions may synergize with antibiotics.

**Methods:**

This work uses knockout mutagenesis, exposure assays, RNA-Seq, promoter reporter assays and ELISA.

**Results:**

We showed that signals from LAB secretions are transduced by 2 histidine sensor kinases and RNA-Seq analysis was performed on gastric cells and on LAB-exposed HP in stomach-mimicking conditions to identify the pathways that lead to reduced bacterial virulence and reduced inflammation. Also, the effect of LAB on HP adherence to and invasion of gastric cells was investigated, and LAB-specific transcriptional effects are being investigated via luminescence reporter assays. Finally, using metabolomics and proteomics we identified candidate active molecules, which will be validated using commercial compounds and knockout mutagenesis, along with fractionation of the most active LAB secretions

**Conclusions:**

This work highlights the variability between LAB strains and provides mechanistic insights to explain the benefits of well-characterized probiotics such as Lacidofil^®^ in anti-HP treatment or prophylaxis. Adjuvant LAB secretions could benefit the ~6 million patients who develop gastric ulcers or cancers each year worldwide due to HP infection, also saving billions of annual health care costs.

**Funding Agencies:**

CIHRWestern University and MITACS

